# Modulation of endogenous pathways enhances bioethanol yield and productivity in *Escherichia coli*

**DOI:** 10.1186/1475-2859-11-145

**Published:** 2012-11-04

**Authors:** Neha Munjal, Anu Jose Mattam, Dibyajyoti Pramanik, Prem Shankar Srivastava, Syed Shams Yazdani

**Affiliations:** 1 Synthetic Biology and Biofuel Group, International Centre for Genetic Engineering and Biotechnology (ICGEB), Aruna Asaf Ali Marg, New Delhi, 110067, India; 2 Centre for Biotechnology, Jamia Hamdard, Hamdard Nagar, New Delhi, 110062, India

**Keywords:** *Escherichia coli*, Endogenous pathways, Promoter engineering, Pyruvate dehydrogenase, Acetate kinase, Ethanol

## Abstract

**Background:**

*E. coli* is a robust host for various genetic manipulations and has been used commonly for bioconversion of hexose and pentose sugars into valuable products. One of the products that *E. coli* make under fermentative condition is ethanol. However, availability of limited reducing equivalence and generation of competing co-products undermine ethanol yield and productivity. Here, we have constructed an *E. coli* strain to produce high yield of ethanol from hexose and pentose sugars by modulating the expression of pyruvate dehydrogenase and acetate kinase and by deleting pathways for competing co-products.

**Results:**

The availability of reducing equivalence in *E. coli* was increased by inducing the expression of the pyruvate dehydrogenase (PDH) operon under anaerobic condition after replacement of its promoter with the promoters of *ldhA*, *frdA, pflB*, *adhE* and *gapA*. The SSY05 strain, where PDH operon was expressed under *gapA* promoter, demonstrated highest PDH activity and maximum improvement in ethanol yield. Deletion of genes responsible for competing products, such as lactate (*ldhA*), succinate (*frdA*), acetate (*ack*) and formate (*pflB*), led to significant reduction in growth rate under anaerobic condition. Modulation of acetate kinase expression in SSY09 strain regained cell growth rate and ethanol was produced at the maximum rate of 12 mmol/l/h from glucose. The resultant SSY09(pZSack) strain efficiently fermented xylose under microaerobic condition and produced 25 g/l ethanol at the maximum rate of 6.84 mmol/l/h with 97% of the theoretical yield. More importantly, fermentation of mixture of glucose and xylose was achieved by SSY09(pZSack) strain under microaerobic condition and ethanol was produced at the maximum rate of 0.7 g/l/h (15 mmol/l/h), respectively, with greater than 85% of theoretical yield.

**Conclusions:**

The *E. coli* strain SSY09(pZSack) constructed via endogenous pathway engineering fermented glucose and xylose to ethanol with high yield and productivity. This strain lacking any foreign gene for ethanol fermentation is likely to be genetically more stable and therefore should be tested further for the fermentation of lignocellulosic hydrolysate at higher scale.

## Background

We are largely dependent upon fossil fuels for fulfilling our energy requirement [1]. Fuels from renewable sources, such as agricultural and forest residues, hold promise in reducing our dependence on fossil fuel without competing with food. The agricultural and forestry waste mostly consist of lignocellulose, which is made-up of highly structured cellulose surrounded by hemicellulose and lignin [2]. In principle, it is possible to breakdown lignocellulose into the monosaccharides and ferment them into ethanol. However, cost associated with this process is a major hurdle in terms of commercial application [3]. One of the key advancement in the economy of ethanol production from lignocellulosic biomass will be to efficiently ferment both hexose and pentose sugars released after hydrolysis of lignocellulose into ethanol. Unfortunately, the conventional microorganisms used for ethanol fermentation, e.g., *Saccharomyces cerevisiae* and *Zymomonas mobilis*, do not have the capability to utilize pentose sugars [4]. Attempts have been made to transfer genes for pentose degradation pathway from other organisms into *S. cerevisiae*[5] and *Z. mobilis*[6]. However, the disadvantages associated with foreign gene expression at large scale like instability, toxicity, containment, etc., prevent its wide usage. *Escherichia coli*, on the other hand, has the ability to ferment both hexose and pentose sugars and is being used to produce ethanol by various genetic manipulation [4]. The genetic manipulation of *E. coli* that does not involve introduction of foreign gene has been attempted with some successes and these technologies certainly have advantages in the long-term genetic stability of the engineered strain [7,8].

**Figure 1:**
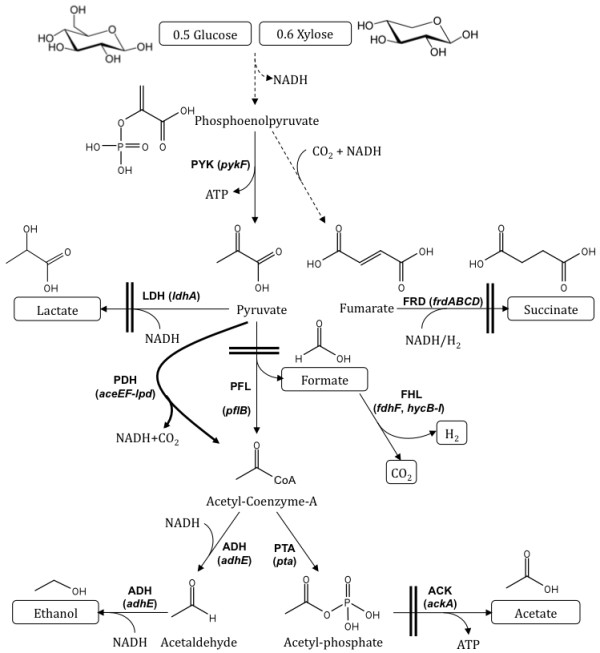
**Central metabolic pathways of *E. coli *functional under anaerobic condition during glucose and xylose fermentation.** Relevant genes and corresponding enzymes are shown. Pyruvate dehydrogenase (PDH) operon was expressed under anaerobic condition via promoter replacement and is represented as thick line. The competing pathways to ethanol were blocked as shown by two parallel bars. Broken arrows represent multiple reactions of a pathway. Extracellular metabolites are placed in boxes. Abbreviations are as follows: ADH, acetaldehyde/alcohol dehydrogenase; ACK, acetate kinase; FHL, formate hydrogen-lyase; FRD, fumarate reductase; LDH, lactate dehydrogenase; PDH, pyruvate dehydrogenase; PFL, pyruvate formate-lyase; PTA, phosphate acetyltransferase; PYK, pyruvate kinase.

Under anaerobic condition, *E. coli* produces ethanol through a pathway that involves pyruvate formate lyase (PFL), which converts pyruvate into acetyl-CoA and formate (Figure 1) [9]. However, this pathway is not redox balanced because in the process of metabolizing one mole of glucose into ethanol, four moles of NADH are consumed while only two moles of NADH are produced (Reaction (i)-(iii)).

(1) $$\text{Glucose}+\text{2 ADP}+{\text{2 NAD}}^{+}\overset{\text{Glycolysis}}{\to }2\;\text{Pyruvate}+\text{2 ATP}+\text{2 NADH}$$

(2) $$2\;\text{Pyruvate}\overset{\text{PFL}}{\to }2\;\text{Acetyl}-\text{CoA}+\text{2 Formate}$$

(3) $$2\;\text{Acetyl}-\text{CoA}+\text{4 NADH}\overset{\text{ADH}}{\to }2\;\text{Ethanol}+{\text{4 NAD}}^{+}$$

This redox imbalance would negatively impact the yield of ethanol. However, there is an alternate pathway exists where converting glucose into ethanol or butanol is a redox balance process. Here pyruvate in converted into acetyl-CoA and CO_2_ via pyruvate dehydrogenase complex (PDH) and in the process one molecule of NADH is produced (Reaction (iv)) [10].

(4) $$2\;\text{Pyruvate}+{\text{2 NAD}}^{+}\overset{\text{PDH}}{\to }2\;\text{Acetyl}-\text{CoA}+2{\text{CO}}_{2}+2\;\text{NADH}$$

However, expression of PDH is repressed under anaerobic condition and remains active in the aerobically growing cells [10]. To activate the expression of PDH under anaerobic condition, the promoter of PDH should be replaced with the one that is highly active under anaerobic condition. One such study showed that replacing PDH promoter with PFL promoter has enhanced the expression of PDH under anaerobic condition and increased the yield of ethanol [8]. However, ethanol productivity was significantly lower in this study indicating sub-optimal flux through the PDH pathway.

In the study reported here, we have performed a systematic study of PDH expression under anaerobic condition by replacing its promoter with a number of promoters of the genes that are expressed at high level under anaerobic condition. A novel promoter-PDH operon combination was selected based on higher PDH enzyme activity and bioalcohol production and deletion mutants were generated to stop carbon flow to the competing byproducts. With the knowledge gained from the growth rate of various deletion mutants, we found a new way to improve cell growth and ethanol productivity by modulating expression of acetate kinase in the engineered cells. We further demonstrate that the engineered *E. coli* strain grown under microaerobic condition utilized xylose with the yield higher than reported before [7,8]. Furthermore, to our knowledge we show for the first time fermentation of mixture of glucose and xylose into ethanol by the engineered *E. coli* strain without having any foreign gene for the ethanol production.

## Results and discussion

### Promoter replacement of pyruvate dehydrogenase (PDH) operon enhances its activity and ethanol yield under anaerobic condition

Ethanol production in *E. coli* through pyruvate formate lyase (PFL) pathway is short of reducing equivalent to achieve a theoretical maximum yield via fermentation of pentose and hexose sugars (Figure 1). Therefore, wild type *E. coli* typically produces mixed acids under fermentative condition with only fraction of carbon goes towards ethanol. The redox balance for homoethanol production may be achieved upon optimal activation of pyruvate dehydrogenase (PDH) pathway under anaerobic condition (Figure 1). The operon encoding PDH complex is usually repressed under anaerobic condition through global repressor binding to its promoter. To prevent this repression, we decided to replace the promoter of PDH operon with the promoter of the genes known to express under anaerobic condition. In the absence of information in the literature regarding relative strength of promoters of the genes expressed under anaerobic condition, we selected promoters of five genes, *frdA*, *ldhA*, *pflB*, *adhE* and *gapA*, and replaced native promoter of PDH operon as mentioned in the methods section. The resultant transformants were verified by internal template based and external host chromosome based primers and were designated as SSY01 to SSY05 for P_ldhA_PDH, P_frdA_PDH, P_pflB_PDH , P_adhE_PDH and P_gapA_PDH promoters, respectively (Table 1).

**Table 1:** Strains, plasmids and primers used in the study

**Name**	**Description**	**Reference or Source**
**Strains**		
*E. coli* B	F-	CGSC #2507
SSY01	*E. coli* B, ΔPDH-promoter::*FRT-kan-FRT-ldhA* gene promoter; promoter of *pdh* gene replaced with promoter of *ldhA* gene	This study
SSY02	*E. coli* B, ΔPDH-promoter::*FRT-kan-FRT-frdA* gene promoter	This study
SSY03	*E. coli* B, ΔPDH-promoter::*FRT-kan-FRT-pflB* gene promoter	This study
SSY04	*E. coli* B, ΔPDH-promoter::*FRT-kan-FRT-adhE* gene promoter	This study
SSY05	*E. coli* B, ΔPDH-promoter::*FRT-kan-FRT-gapA* gene promoter	This study
SSY06	SSY05 Δ*ldhA*::*FRT-kan-FRT;* deletion mutant for *ldhA* gene in SSY05 host	This study
SSY07	SSY06 Δ*frdA*::*FRT-kan-FRT;* deletion mutant for *frdA* gene in SSY06 host	This study
SSY08	SSY07 Δ*ackA*::*FRT-kan-FRT;* deletion mutant for *ackA* gene in SSY07 host	This study
SSY09	SSY08 Δ*pflB*::*FRT-kan-FRT;* deletion mutant for *pflB* gene in SSY08 host	This study
**Plasmids**		
pUC19	*bla*, cloning vector	
pKD4	*bla,* FRT-kan-FRT	CGSC #7632
pKD46	*bla, γ β exo* (red recombinase), temperature-conditional replicon	CGSC #7739
pCP20	*bla, flp*, temperature-conditional replicon	CGSC #7629
pSSY01	FRT-kan-FRT sequence from pKD4 was cloned into pUC19 at *Eco*RI and *Bam*HI sites	This study
pSSY02	*ldh*A gene promoter from *E. coli* B was cloned into pSSY01 at *Bam*HI and *Hind*III site	This study
pSSY03	*frd*A gene promoter from *E. coli* B was cloned into pSSY01 at *Bam*HI and *Hind*III sites	This study
pSSY04	*pfl*B gene promoter from *E. coli* B was cloned into pSSY01 at *Bam*HI and *Hind*III sites	This study
pSSY05	*adh*E gene promoter from *E. coli* B was cloned into pSSY01 at *Bam*HI and *Hind*III sites	This study
pSSY06	*gap*A gene promoter from *E. coli* B was cloned into pSSY01 at *Bam*HI and *Hind*III sites	This study
pZSblank	P_LtetO1_ expression vector, pSC101*origin, Cm^R^	[11]
pZS*mcs	multiple cloning site derived from pET28a(+) cloned in pZSblank	This study
pZSack	*ack* gene cloned in pZS*mcs vector	This study
**Primers**		
FRT-kan-FRT-F	GGAGAGAATTCGTGTAGGCTGGAGCTGCTTC	This study
FRT-kan-FRT-R	GGAGAGGATCCATATGAATATCCTCCTTAG	This study
*ldh*A promoter-F	TCGGGATCCGCAAGCTGACAATCTCCC	This study
*ldh*A promoter-R	ACTCAAGCTTAAGACTTTCTCCAGTGATGTTG	This study
*frd*A promoter-F	TGCGGATCCATCAAACAGCGGTGGGCAG	This study
*frd*A promoter-R	CCCAAGCTTGACATTCCTCCAGATTGTTT	This study
*pfl*B promoter-F	TCGGGATCCAACCATGCGAGTTACGGGCCTATAA	[8]
*pfl*B promoter-R	CCCAAGCTTGTGCCTGTGCCAGTGGTTGCTGTGA	This study
*adh*E promoter-F	CGCGGATCCCCGGATAATGTTAGCCATAA	This study
*adh*E promoter-R	CCCAAGCTTAATGCTCTCCTGATAATGTTA	This study
*gap*A promoter-F	CGCGGATCCGATTCTAACAAAACATTAACAC	This study
*gap*A promoter-R	CCCAAGCTTATATTCCACCAGCTATTTGT	This study
H1	*CTCCTTTCCTACGTAAAGTCTACATTTGTGCATAGTTACAACTTT*GTGTAGGCTGGAGCTGCTTC	[8]
H2_*ldhA*	*GCGAGTTTCGATCGGATCCACGTCATTTGGGAAACGTTCTGACAT*AAGACTTTCTCCAGTGATGTTG	This study
H2_adhE	*GCGAGTTTCGATCGGATCCACGTCATTTGGGAAACGTTCTGACAT*AATGCTCTCCTGATAATGTT	This study
H2_gapA	*GCGAGTTTCGATCGGATCCACGTCATTTGGGAAACGTTCTGACAT*ATATTCCACCAGCTATTTGT	This study
H2_frdA	*GCGAGTTTCGATCGGATCCACGTCATTTGGGAAACGTTCTGACAT*GACATTCCTCCAGATTGTTT	This study
H2_pflB	*GCGAGTTTCGATCGGATCCACGTCATTTGGGAAACGTTCTGACAT*GTAACACCTACCTTCTGTTG	[8]
	CTGTGATATAGAAGAC	
v-PDH-F	TGCATGGTTGAAGATGAGTTG	This study
v-PDH-R	TGATGTAGTTGCTGATACCTG	This study
pET28mcs-F	CGGGATCCGAATTCGAGCTC	This study
pET28mcs-R	CTGACGCGTGTTAACAGCTTCCTTTCGGGCTTTG	This study
pZS-ack-F	GATCGGATCCATGTCGAGTAAGTTAGTACTGGT	This study
pZS-ack-R	TCGAGTCGACTCAGGCAGTCAGGCGGCTC	This study

Note: Homologous region for recombination is in italics and the enzyme sites are underlined.

**Figure 2:**
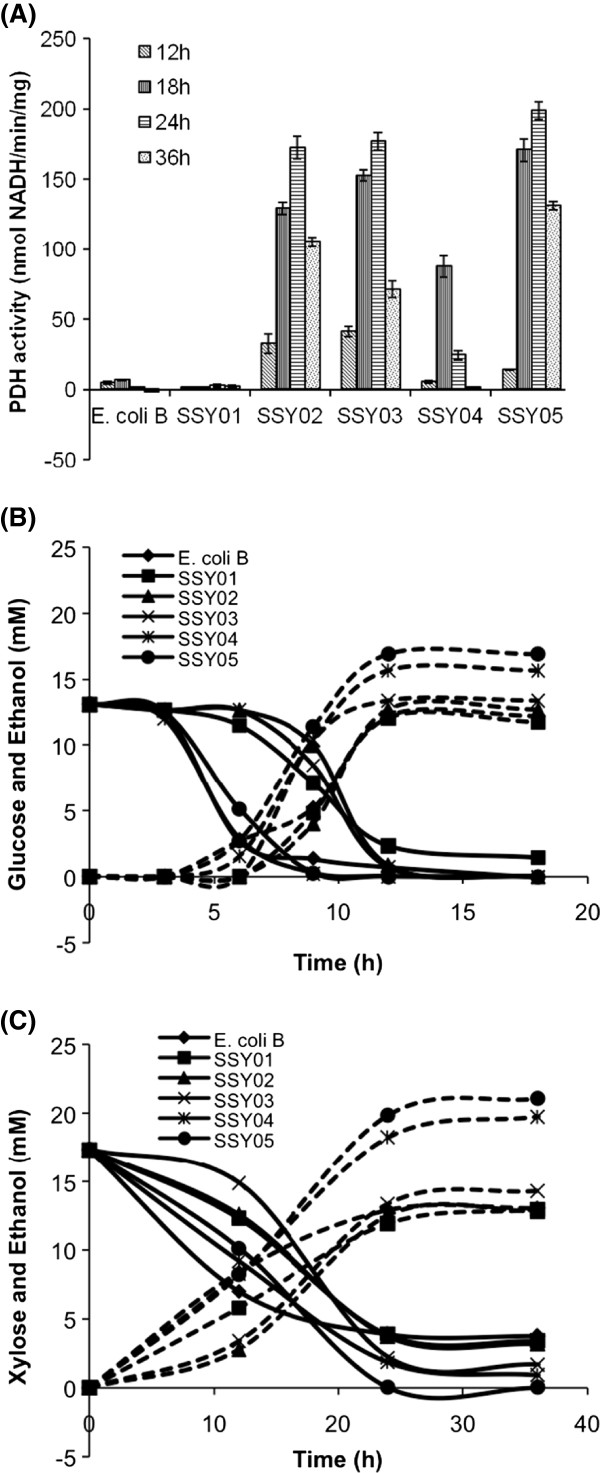
**Functional characterization of promoter engineered *E. coli *B strains (SSY01-05, see Table**1**for genotype).** Effect of PDH operon promoter replacement on **(A)** pyruvate dehydrogenase activity and **(B)** ethanol production was monitored. Cells were grown anaerobically in completely filled Hungate tubes and were harvested and permeabilized to measure PDH activity. The supernatant of the culture was used to analyze metabolite concentration via HPLC. Strain description for changed PDH promoter: SSY01 – P_ldhA_PDH, SSY02 –P_frdA_PDH, SSY03 – P_pflB_PDH, SSY04 – P_adhE_PDH, SSY05 – P_gapA_PDH.

The engineered cells were grown under anaerobic condition in defined medium for different time intervals and used for measuring pyruvate dehydrogenase (PDH) activity. The results indicated maximum PDH activity between 18–24 h of anaerobic growth (Figure 2A). Except SSY01 strain (P_ldhA_PDH), all promoter engineered strains showed significant improvement in PDH activity over wild type strain. This result was intriguing since *pdh* gene in *Geobacillus thermoglucosidasius* under the control of *ldh* promoter was shown to have positive effect on ethanol production [12], indicating higher flux through PDH pathway in this strain due to higher PDH activity. We therefore compared ethanol production capabilities of all the engineered strains to assess flux towards PDH pathway.

The engineered strains SSY01 to SSY05 along with wild type *E. coli* B were grown in defined medium in filled Hungate tube with 2.5 g/l of either glucose or xylose as carbon source and cultures were analyzed for production of ethanol at different time intervals. Most engineered strains utilized complete 13.9 mM glucose in 12 h and produced ethanol in the range of 11.7 mM (for SSY01) to 17 mM (for SSY05) as compared to *E. coli* B which produced 12.2 mM ethanol. It took 24 h for the *E. coli* strains to utilize xylose (17 mM) with 0% (for SSY05) to 20% (for *E. coli* B and SSY01) residual sugar left at the end. Ethanol from xylose was produced in the range of 13 mM (for SSY01) to 21 mM (for SSY05) as compared to wild type *E. coli* B that produced 13 mM ethanol. These observations indicated that SSY01 with P_ldh_PDH genotype behaved similar to wild type *E. coli* in terms of ethanol production as against *Geobacillus thermoglucosidasius* where it improved ethanol production [12]. Possible explanation of these observations could be that *ldh* promoter in *E. coli* was either relatively weaker or regulated through complex mechanism under anaerobic condition. This hypothesis needs further exploration. Other engineered strains showed higher ethanol production as compared to the wild type strain. SSY05 performed best among all the engineered stains and therefore was considered for further strain improvement. Our approach of first optimizing the flux towards the PDH pathway through promoter engineering in the wild type strain before any deletion in the competing pathway, as against the previous report where the flux through PDH pathway was enforced by first deleting the competing pathway [8], had an advantage of finding optimal flux through PDH pathway even in presence of competing PFL pathway that is considered essential under anaerobic condition [9]. This was likely to lower the adverse impact on cell growth upon *pflB* deletion.

### Deletion of competing pathways improves ethanol yield in the engineered SSY05 strain

**Figure 3:**
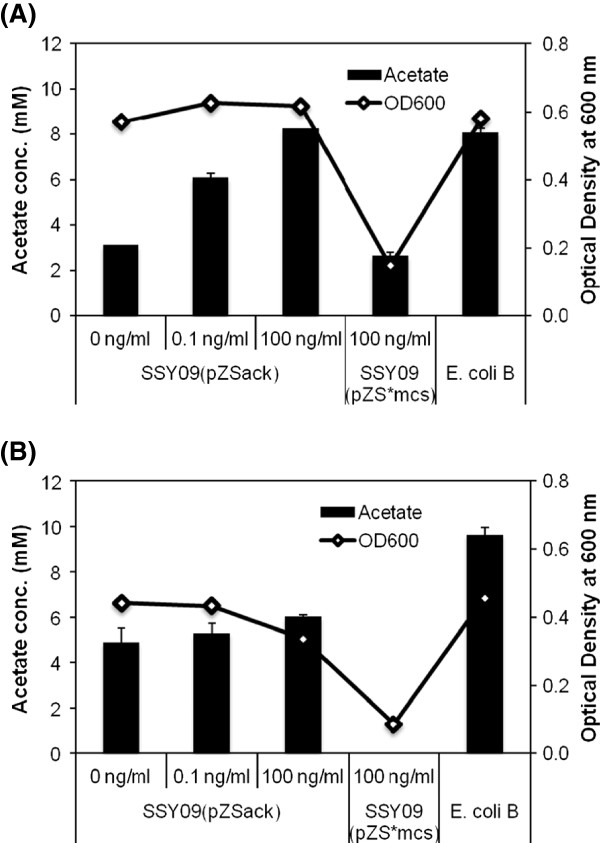
**Improvement in cell growth upon modulation of expression of *ack *gene in the engineered SSY09 strain.** SSY09 strain having plasmid pZSack was grown in Hungate tube completely filled with defined media + 2.5g/l glucose **(A)** or 2.5g/l xylose **(B)** at 37°C for 24 hr. Acetate kinase expression was induced with 0, 0.1, 100 ng/ml of anhydrotetracycline. *E. coli* B and SSY09 bearing pZS*mcs plasmid were used as positive and negative control, respectively. Results indicate improvement in growth of PZSack transformed cells as compared to control plasmid and less acetate production as compared to wild type strain. Strain description: SSY09 - P_gapA_PDH Δ*ldhA* Δ*frdA* Δ*ack* Δ*pflB*.

Though the promoter engineered SSY05 strain exhibited significant enhancement in the ethanol level as compared to the wilde type strain, it still produced considerable amount of competing co-products such as lactate, succinate, acetate and formate (Table 2). To further improve ethanol yield, we introduced deletion in the genes for lactate dehydrogenase (*ldhA*), fumarate reductase (*frdA*), acetate kinase (*ack*) and pyruvate formate lyase (*pflB*) responsible for the formation of lactate, succinate, acetate and formate, respectively, to obtain SSY06 (P_gapA_ PDH Δ*ldhA*), SSY07 (P_gapA_ PDH Δ*ldhA* ΔfrdA), SSY08 (P_gapA_ PDH Δ*ldhA* ΔfrdA Δack) and SSY09 (P_gapA_ PDH Δ*ldhA* ΔfrdA Δack ΔpflB) strains (Table 1). When grown in defined medium under anaerobic condition, SSY06 and SSY07 grew normally (Table 2), SSY08 grew very slowly and SSY09 did not grow at all. This observation indicated that deletion of *ack* had deleterious impact on cell growth, possibly due to corresponding depletion of ATP pool, and further deletion of *pflB* had a cumulative effect on adverse impact even after the expression of parallel PDH pathway. Deletion of *ack* and *pflB* leading to adverse impact on cell growth under anaerobic condition has also been observed earlier [9,13]. To regain the cell growth, we introduced *ack* gene through a very low copy plasmid in SSY09 strain. We found significant improvement in cell growth upon transformation with the plasmid containing *ack* gene (pZSack) as compared to the strain transformed with the control plasmid (pZS*mcs) even without addition of an inducer, indicating minor leaky expression of *ack* gene (Figure 3A and 3B). Acetate level in uninduced SSY09(pZSack) strain was less than 50% of the wild type strain. We further tested ethanol-producing capability of the engineered strains at the bioreactor level under controlled environmental condition.

**Table 2:** Fermentation parameters for cell growth, sugar utilization and product synthesis at the bioreactor level

**Sugar**	**Strain**	**Medium+Sugar Conc.**^**a**^	**Product Yield (mmol per mmol sugar)**						**% Theoretical yield of ethanol**^b^	**Max ethanol productivity**^**C**^	
			**Cells**	**Succinate**	**Lactate**	**Formate**	**Acetate**	**Ethanol**		**Specific (mmol/g/h)**	**Volumetric (mmol/l/h)**
Glucose	*E. coli* B	Defined medium+20 g/l	0.36	0.11	0.42	1.19	0.51	0.65	32	4.82	4.72
	SSY05	Defined medium+20 g/l	0.40	0.14	0.13	0.98	0.42	0.84	42	5.76	6.19
	SSY06	Defined medium+20 g/l	0.42	0.14	0.02	1.15	0.53	0.96	48	6.42	6.91
	SSY07	Defined medium+20 g/l	0.46	0.01	0.03	1.16	0.46	1.08	54	4.85	5.97
	SSY09(pZSack)	Defined medium+20 g/l	0.25	0.00	0.00	0.00	0.12	1.67	83	4.00	1.62
	*E. coli* B	LB medium+50 g/l	0.23	0.07	0.78	0.57	0.37	0.31	16	6.15	3.06
	SSY09(pZSack)	LB medium+50 g/l	0.16	0.01	0.01	0.03	0.07	1.89	95	20.03	12.34
Xylose	*E. coli* B	Defined medium+20 g/l	0.34	0.17	0.00	1.21	0.80	0.61	36	2.46	1.54
	SSY09(pZSack)	Defined medium+20 g/l	0.24	0.01	0.02	0.00	0.14	1.09	68	6.15	1.96
	*E. coli* B	LB medium+50 g/l	0.20	0.10	0.05	0.30	0.70	0.79	47	3.13	1.90
	SSY09(pZSack)	LB medium+50 g/l	0.18	0.02	0.01	0.03	0.26	0.91	55	1.57	1.67
	SSY09(pZSack)	LB medium+50 g/l (Microaerobic, pH 6.3)	0.40	0.01	0.01	0.01	0.17	1.63	97	5.72	6.84
Glucose+Xylose	SSY09 (pZSack)	LB medium+50 g/l (Microaerobic, pH 6.3)	0.64	0.01	0.00	0.00	0.04	1.61	85	5.43	14.94

^a^ Growth condition in the fermentor is described in materials and method section.

^b^ % Theoretical yield of ethanol was calculated by considering theoretical maximum yield of 2 mmol of ethanol per mmol of glucose and 1.67 mmol of ethanol per mmol of xylose as 100%.

^c^ Maximum specific (mmol ethanol per gram of cells per hour) and volumetric (mmol per liter of culture per hour) productivity of ethanol were calculated by accounting the interval at which maximum substrates were consumed and maximum products and cell biomass were formed.

### Comparison of engineered strains for ethanol production at the bioreactor level in defined medium

**Figure 4:**
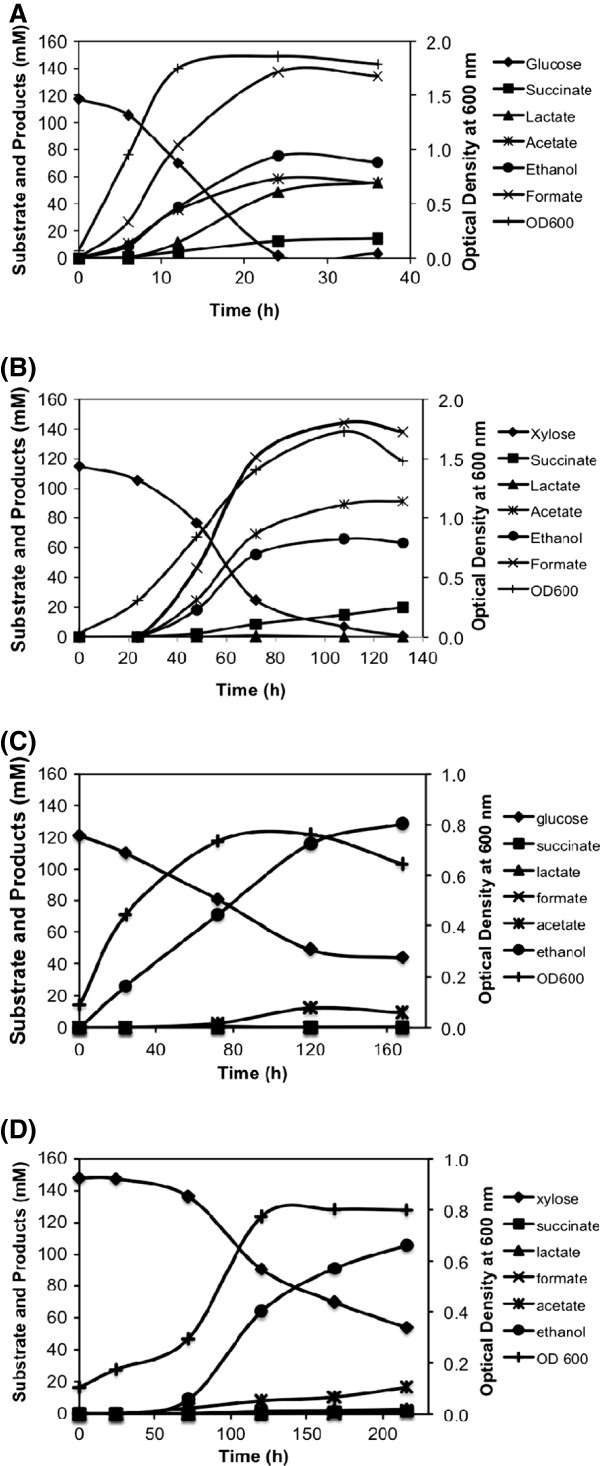
**Fermentation profiles of *E. coli *B (A and B) and SSY09(pZSack) (C and D) grown in the bioreactor in defined medium with glucose (A and C) and xylose (B and D) as carbon source.** Competing products of ethanol are produced at significant level during fermentation of both glucose and xylose in *E. coli* B while SSY09(pZSack) primarily produced ethanol. SSY09(pZSack) - P_gapA_PDH Δ*ldhA* Δ*frdA* Δ*ack* Δ*pflB* (pZSack).

When grown in a bioreactor, wild type *E. coli* B produced ethanol at the yield of 0.65 and 0.61 mmol per mmol of glucose and xylose, respectively, in defined medium under anaerobic condition as against the theoretical maximum yield of 2 and 1.67 mmol per mmol of these sugars due to generation of competing co-products (Figure 4A and 4B, Table 2). The promoter engineered SSY05 strain showed 10% higher ethanol yield as compared to wild type strain, indicating favourable redox balance towards ethanol production (Table 2). Successive deletion made in the competing pathways to generate SSY06, SSY07 and SSY09(pZSack) strains resulted in corresponding increase in ethanol and decrease in co-product yield (Table 2). SSY09(pZSack) strain grown in defined medium with 20 g/l glucose or xylose at the bioreactor level produced ethanol at 83% or 68% of theoretical maximum yield, respectively (Figure 4C and 4D, Table 2). However, ~25% of substrate remained unutilized at ~200 hrs of fermentation and ethanol was produced at the low volumetric productivity of 1.6-1.9 mmol/l/h. This observation indicated that the engineered PDH pathway possibly could not fully complement the loss the PFL pathway.

### Comparison of engineered strains for ethanol production at the bioreactor level in complex medium

**Figure 5:**
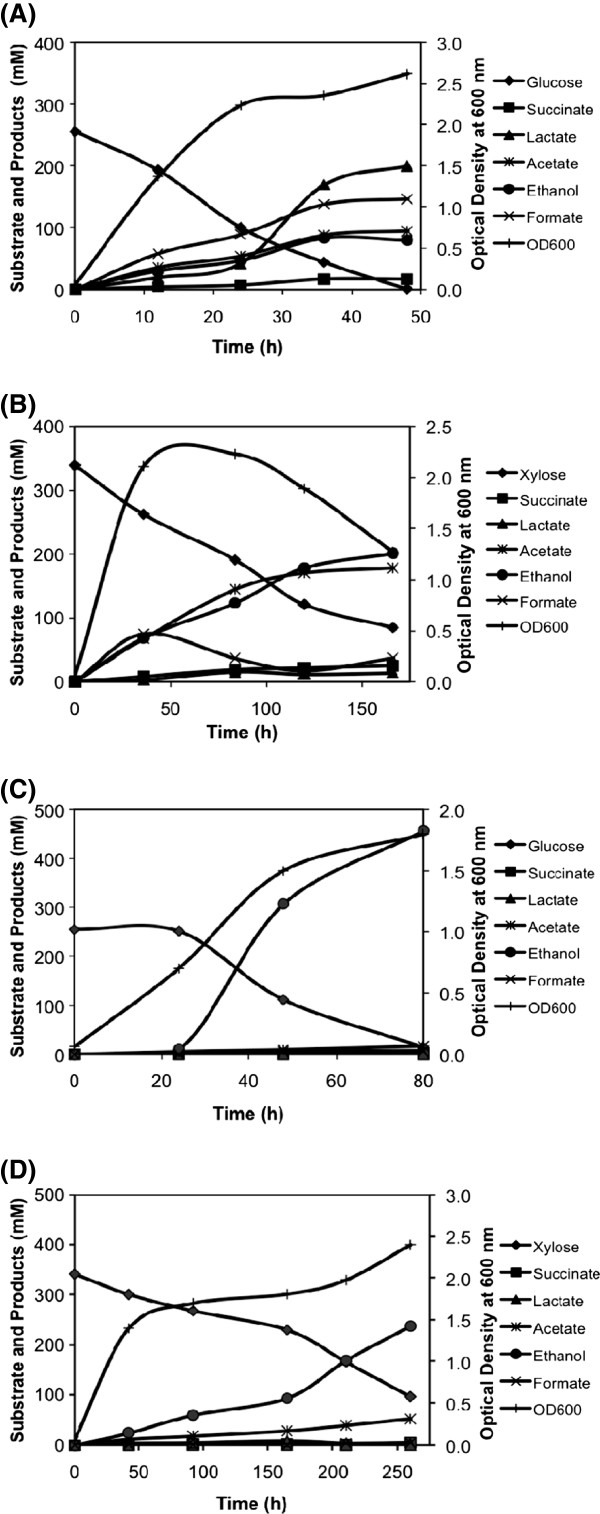
**Fermentation profile of *E. coli *B (A and B) and SSY09(pZSack) (C and D) grown in the bioreactor in complex medium with glucose (A and C) and xylose (B and D) as carbon source.** Only small fraction of carbon has been used by the *E. coli* B cells to produce ethanol. SSY09(pZSack) strain utilized glucose and produced ethanol at a significantly high rate. Xylose utilization rate, however, was still slow. Strain description: SSY09(pZSack) - P_gapA_PDH Δ*ldhA* Δ*frdA* Δ*ack* Δ*pflB* (pZSack).

To improve the growth, substrate utilization and ethanol production rate, we grew the engineered cells in LB medium with 50 g/l substrate. The wild type strain produced lactate and acetate as major metabolic products with ethanol produced only at the yield of 0.31 and 0.79 mmol per mmol glucose and xylose, respectively (Figure 5A and 5B, Table 2). Remarkable improvement in growth rate was observed in the case of SSY09(pZSack) strain when grown in complex medium. The growth profile indicated that complex media served as nutrient supplement for achieving initial cell growth, and sugar consumption occurred after the growth cycle (Figure 5C and 5D). This strategy allowed the cells to overcome the growth limitation arose due to *pflB* deletion. Ethanol was produced at the rate of 12.34 mmol/l/h with 95% of the theoretical yield using glucose as carbon source (Figure 5C, Table 2). The final ethanol concentration achieved was 21 g/l (457 mM) from 46 g/l (255 mM) glucose in 80 h (Figure 5C).

**Figure 6:**
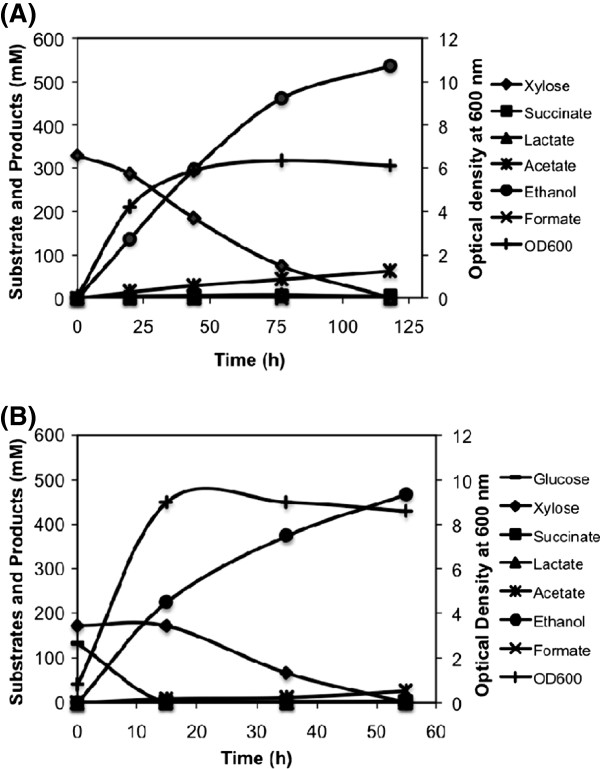
**Fermentation profile of SSY09(pZSack) strain grown under microaerobic condition in the bioreactor in complex medium with (A) xylose and (B) mixture of glucose and xylose as carbon source.** The profile indicated efficient utilization of xylose and mixture of glucose and xylose under microaerobic condition and production of ethanol with high yield and productivity. Strain description: SSY09(pZSack) - P_gapA_PDH Δ*ldhA* Δ*frdA* Δ*ack* Δ*pflB* (pZSack).

The xylose utilization rate, however, was still slow in the SSY09(pZSack) strain with 25% xylose remained unutilized after 260 h of fermentation and ethanol produced only at the rate of 1.67 mmol/l/h (Figure 5C, Table 2). This was because xylose fermentation into ethanol leads to generation of only 0.67 ATP per xylose as against glucose fermentation where 2 ATPs per glucose are generated. There was a slight increase in xylose utilization rate when pH of the cultivation was maintained at 6.3 (data not shown), possibly due to higher activity of a xylose/proton symporter [14]. To further enhance the growth rate of the cells, we introduced a microaerobic condition by passing compressed air in the headspace of bioreactor at a very slow flow rate as mentioned in the methods section. Microaerobic condition would allow extra ATP for cell growth through partial activation of TCA cycle. Cell growth and xylose utilization rate improved significantly with 50 g/l xylose utilized in 115 h and 25 g/l ethanol produced at the rate of 6.84 mmol/l/h with 97% of the maximum theoretical yield (Figure 6A). This yield of ethanol from xylose was higher than those reported in the literature from the engineered *E. coli* without the foreign genes [7,8].

We further tested utilization of mixture of glucose and xylose at 25 g/l each under micro-aerobic condition and found complete utilization of sugars in 55 hrs (Figure 6B). As evident from the fermentation profile, glucose was the preferred substrate and got utilized first in 15 h followed by xylose utilization in next 40 h. Ethanol yield from glucose and xylose was close to 0.43 g per g sugar (85% of the maximum theoretical yield) and ethanol was produced at the rate of 14.94 mmol/l/h (0.7 g/l/h) during glucose utilization phase and 8.17 mmol/l/h (0.38 g/l/h) during xylose utilization phase (Figure 6B). This rate of ethanol production from mixture of glucose and xylose was close to that of- recombinant *E. coli* KO11 strain that produced ethanol at overall 0.34 g/l/h from mixture of 10g/l glucose and 40 g/l xylose [15] and at 0.72 g/l/h h in the first 48 h from mixture of 70 g/l glucose and 30 g/l/h xylose of fermentation [16]. None of the reports published before for the engineered *E. coli* without the foreign genes demonstrated utilization of mixture of glucose and xylose [7,8].

The *E. coli* SSY09(pZSack) strain engineered for ethanol production in this study certainly has advantage over the other engineered *E. coli* strains such as KO11 for not having any foreign genes responsible for ethanol production. *E. coli* KO11 has been found to lose its ethanologenicity progressively when cultivated on hemicellulosic sugars in the chemostat culture, possibly due to the genetic instability [17]. Since *E. coli* SSY09(pZSack) does not have any foreign genes for ethanol production, its ethanologenic property is expected to be stable for much longer generation and therefore this strain should be considered for further studies to evaluate ethanol production from lignocellulosic hydrolysates.

## Conclusions

We replaced promoter of pyruvate dehydrogenase operon (PDH) in *E. coli* with promoters of various genes expressed under anaerobic condition and shown that PDH expression and ethanol yield was maximum under anaerobic condition when its promoter was replaced with *gapA* promoter. Deletion of pathways for competing products further increased the ethanol yield. However, there was significant drop in cell growth rate. Modulating expression of acetate kinase helped restoring the cell growth rate and improved ethanol productivity significantly. Microaerobic condition further improved the growth rate of the cells on both glucose and xylose. The strain reported here following engineering of endogenous pathway is likely to be genetically more stable and call for further study to evaluate ethanol production from hydrolysate of lignocellulosic biomass.

## Methods

### Bacterial strains, plasmids and genetic methods

List of bacterial strains, plasmids and primers used in the study has been provided in Table 1. *E. coli* DH5α strain (Invitrogen) was used for performing all the cloning work and *E. coli* B (Coli Genetic Stock Centre (CGSC), Yale University, USA) was used as parent strain for all the genomic manipulations. Recombinant DNA techniques were performed according to standard procedures [18]. Restriction endonuclease and T4 DNA ligase were procured from New England Biolabs and DNA purification was performed using Qiagen kit. Custom oligonucleotides (primers) were synthesized from Sigma-Aldrich for PCR amplifications. DNA fragments were amplified by Phusion High Fidelity polymerase (Finnzymes) for cloning and template preparation for homologous recombination and *Taq* DNA polymerase (Bangalore Genei) was used for performing verification PCR of the engineered strains. Plasmids pKD4, pKD46 and pCP20 (CGSC, USA) were used as the source of FRT-kan-FRT fragment, lambda Red recombinase and flippase, respectively, for performing genetic manipulation*.*

For replacement of PDH operon promoter in the *E. coli* B genome, following procedure was adopted. FRT-kan-FRT sequence from pKD4 was amplified using FRT-kan-FRT-F and FRT-kan-FRT-R primers (Table 1), digested with *Eco*RI and *Bam*HI and ligated to the corresponding restriction endonuclease sites of pUC19 plasmid to generate the plasmid pSSY01. Promoters of the genes *ldhA, adhE, frdA, gapA* and *pflB*, along with their corresponding ribosome binding sites, were amplified from *E. coli* B genomic DNA using their respective primers listed in Table 1. The PCR products obtained were digested with *Bam*HI and *Hind*III and ligated at the 3’ end of FRT-kan-FRT in pSSY01 to produce plasmids pSSY02-06 (Table 1). A 45 bases homologous sequence for −202 to −157 bp upstream of *pdhR* coding region of PDH operon was added to the 20 bases of 5’ end of FRT-kan-FRT sequence to design primer H1 and a 45 bases homologous sequence corresponding to +1 to +45 coding region of *ace*E of PDH operon was added to 20–22 bases of 3’ end of each promoter to obtain primer H2 (Table 1). PCR was performed with the H1 and H2 primers and corresponding plasmid pSSY02-06 as template under following conditions: 98°C for 2 min, followed by 30 cycles of denaturation at 98°C for 15 sec, annealing at 59°C for 15 sec, extension at 72°C for 2 min and a final extension at 72°C for 10 min. The PCR product was gel eluted, digested with DpnI, re-purified and electroporated (2.5 KV, 25 μF and 200 Ω) into *E. coli* B carrying pKD46 (grown in LB broth with 1 mM L-arabinose at 30°C till OD_600nm_ reaches ~ 0.3 - 0.4) to replace the promoter, RBS and pdhR gene of pyruvate dehydrogenase (PDH) operon with the heterologous promoter [19]. Transformants were selected on kanamycin LB-agar plates. The engineered strains (SSY01-05) (Table 1) were verified for the PDH promoter replacement by performing two sets of colony PCR, one set using v-PDH-F (−372 bp upstream of *pdhR*) and v-PDH-R (+163 bp downstream of start of coding region of *aceF*) primers to verify native promoter deletion, and second set using forward primer of the heterologous promoter and v-PDH-R to verify introduction of heterologous promoter (data not shown). Before further manipulation, the kanamycin resistance marker gene was removed from the chromosome of the selected strain with the help of FLP recombinase by using the temperature sensitive helper plasmid, pCP20 [19].

Host gene deletions were achieved through P1 transduction method [20] using the single gene knockout Keio strains from CGSC, Yale University, USA [21]. The kanamycin resistant marker gene was removed as described above and the resultant strain was used for sequential rounds of gene knockout.

For the construction of pZSack plasmid, the multiple cloning site (MCS) of pET28a(+) (Novagen) was amplified using pET28mcs-F and pET28mcs-R primers and cloned in pZSblank plasmid [11] to obtain pZS*mcs. The *ack*A gene encoding acetate kinase was amplified from *E. coli* B genome using pZS-ack-F and pZS-ack-R primers, digested with *Bam*HI and *Sal*I and the resultant fragment was ligated into the *Bam*HI-*Sal*I sites of pZS*mcs to produce pZSack. The pZSack plasmid was then electroporated into SSY09 for enhancement of growth rate.

### Media and culture conditions

Bacterial strains were grown in either LB medium or Morpholino-propanesulfonic (MOPS) defined medium [22]. Antibiotics were added as appropriate with ampicillin at 50 μg/ml, kanamycin at 30 μg/ml and chloramphenicol at 34 μg/ml. For checking production of metabolites by the engineered strains in the tube, the strains were grown overnight at 37°C on LB agar plates containing relevant antibiotic and an isolated colony was inoculated in Hungate tube filled until brim (17.5 ml) with 1X MOPS or LB medium supplemented with antibiotics and desired sugar as carbon source. In the study where engineered strains were transformed with pZSack or pZS*mcs plasmid, the cells were grown in the Hungate tube filled with media containing 0, 0.1 or 100 ng/ml of anhydrotetracycline as inducer and 34 μg/ml of chloramphenicol as antibiotic. The tubes were incubated at 37°C under rotating condition and harvested at different intervals. The optical density of the grown culture was recorded at 600 nm and supernatant was saved for metabolite analysis via HPLC as mentioned in analytical methods section.

The engineered strains were cultivated in the bioreactor to evaluate their performance under controlled environment at various stages of manipulation. Primary culture was prepared by incubating an isolated colony from agar plate into 17.5 ml MOPS medium containing 2.5 g/l glucose in Hungate tube for 24 hr at 37°C. In case of SSY09 where *ack* and *pflB* genes were deleted, primary culture was adapted to anaerobic condition in 100 ml medium in a 250 ml flask containing 2.5 g/l glucose or xylose for 48 hr at 37°C in anaerobic chamber (Bactron II, Shel Lab). Appropriate volume of the culture to achieve initial OD_600nm_ of 0.05 in the bioreactor was centrifuged at 4000 rpm for 4 min and re-suspended in fresh medium. The culture was inoculated in one of the six 0.5 L vessels of Biostat Q plus fermentor (Sartorius) containing 350 ml of MOPS or LB medium having appropriate amount of sugar. The vessels were controlled independently at 37°C, 300 rpm and pH 6.8. High purity Argon gas was purged in the medium to create anaerobic environment at a rate of 0.02 L/min. In case of fermentation under microaerobic condition, compressed air was passed in the headspace of the vessel at the rate of 0.02L/min at which dissolved oxygen probe demonstrated zero reading throughout the fermentation. Samples collected from fermentor vessels at various time intervals were used to calculate cell growth, substrate utilization and product synthesis. All fermentations were performed in duplicate and data in the figures represented average of two bioreactor runs.

### Enzyme assay

To check the activity of PDH enzyme under anaerobic conditions, engineered *E. coli* B strains with heterologous PDH promoter along with wild type strain as control were grown in Hungate tubes filled with MOPS medium + 2.5 g/l glucose for 12, 18, 24 and 36 hrs at 37°C. Cells were harvested by centrifugation (5 min, 5000 rpm), washed twice with 9 g/l NaCl and stored as cell pellets at −20°C. Cell pellets were resuspended in 0.1 M potassium phosphate buffer (pH 8.0) to obtain OD_600_ of 10 and were permeabilized with chloroform. The reaction was set-up in 1 ml in the cuvette containing 50 mM potassium phosphate buffer (pH 8.0), 2.0 mM sodium pyruvate, 2.5 mM NAD^+^, 0.2 mM thiamine pyrophosphate, 1.0 mM MgCl_2_, 0.13 mM CoA, 2.6 mM cysteine hydrochloride. Permeabilized cells (25 μl) were added to start the reaction and the pyruvate dehydrogenase activity was measured by detecting change in absorbance at 340 nm (Ultrospec 3100 pro, Amersham Biosciences) [23]. Substrate blank where no sodium pyruvate was added served as control. Enzyme activity was calculated as nmol NADH formed/min/mg of cell protein. A protein content of 50% (wt/wt) with respect to dry cell mass was assumed in these calculations.

### Analytical methods

Extracellular metabolites of the grown culture were determined as follows. Culture of the grown cells was centrifuged at 13,200 rpm for 5 min. The aqueous supernatant was filtered and used for HPLC analysis. The metabolite separation was achieved using the HPLC system (Agilent technologies) attached with Aminex HPX-87 H anion exchange column (Bio-Rad). The filtered and degassed mobile phase (4 mM H_2_SO_4_) was used at a constant rate of 0.3 ml/min with column and RI detector temperatures maintained at 40°C and 35°C, respectively. Standards of the metabolites (Absolute Standards, USA) at 1 g/l were separated on HPLC column and areas obtained were used to calculate metabolite concentration in the text samples. Cell density was measured at an optical density 600 nm (OD_600_) in a spectrophotometer (BioRad). Dry cell mass was calculated by drying cell pellets of defined OD_600_ at 75°C in oven for 20 hr. The OD_600_ of 1.0 corresponded to 0.56 mg dry mass per ml of culture.

The values obtained for cell biomass, substrate utilization and product synthesis were used for calculation of biomass and product yields (mmol/mmol substrate), specific productivity (mmol/gcell/h) and volumetric productivity (mmol/L/h). For calculating biomass yield we used a molecular formula of cells as CH_1.9_O_0.5_N_0.2_ with an average molecular weight of 24.7 [11].

## Competing interest

The authors declare that they have no competing interests.

## Authors’ contributions

NM carried-out all the promoter replacements, NM and AJM performed phage transduction for creating deletion mutants, DP and NM performed fermentation studies, PSS provided intellectual input for the study, SSY conceived and coordinated the study and SSY and NM drafted the manuscript. All authors read and approved the final manuscript.
